# Chitinase 3-like 1 is neurotoxic in primary cultured neurons

**DOI:** 10.1038/s41598-020-64093-2

**Published:** 2020-04-28

**Authors:** Clara Matute-Blanch, Laura Calvo-Barreiro, Iria Carballo-Carbajal, Ricardo Gonzalo, Alex Sanchez, Miquel Vila, Xavier Montalban, Manuel Comabella

**Affiliations:** 1 Servei de Neurologia-Neuroimmunologia, Centre d’Esclerosi Múltiple de Catalunya (Cemcat), Institut de Recerca Vall d’Hebron (VHIR), Hospital Universitari Vall d’Hebron, Universitat Autònoma de Barcelona, Barcelona, Spain; 2 Neurodegenerative Diseases Research Group, Vall d’Hebron Research Institute-Center for Networked Biomedical Research on Neurodegenerative Diseases (CIBERNED), Barcelona, Spain; 3 Statistics and Bioinformatics Unit. Vall d’Hebron Institut de Recerca, Barcelona, Spain; 4 Genetics, Microbiology and Statistics Department, Universitat de Barcelona, Barcelona, Spain; 5 Department of Biochemistry and Molecular Biology, Autonomous University of Barcelona, Barcelona, Spain; 6 Catalan Institution for Research and Advanced Studies (ICREA), Barcelona, Spain; 7 Division of Neurology, St. Michael’s Hospital, University of Toronto, Toronto, ON Canada

**Keywords:** Diagnostic markers, Predictive markers, Prognostic markers, Cell death in the nervous system

## Abstract

Chitinase 3-like 1 (CHI3L1) is known to play a role as prognostic biomarker in the early stages of multiple sclerosis (MS), and patients with high cerebrospinal fluid CHI3L1 levels have an increased risk for the development of neurological disability. Here, we investigated its potential neurotoxic effect by adding recombinant CHI3L1 *in vitro* to primary cultures of mouse cortical neurons and evaluating both neuronal functionality and survival by immunofluorescence. CHI3L1 induced a significant neurite length retraction after 24 and 48 hours of exposure and significantly reduced neuronal survival at 48 hours. The cytotoxic effect of CHI3L1 was neuron-specific and was not observed in mouse immune or other central nervous system cells. These results point to a selective neurotoxic effect of CHI3L1 *in vitro* and suggest a potential role of CHI3L1 as therapeutic target in MS patients.

## Introduction

Recent studies have pointed to a prognostic role of the astrocyte-specific biomarker chitinase 3-like 1 (CHI3L1) in patients with clinically isolated syndrome (CIS)^1,2^. In an initial study conducted by our group, CIS patients were classified according to their conversion to clinically definite multiple sclerosis (MS)^1^. By applying a mass spectrometry-based proteomic approach, CHI3L1 was identified as one of the most differentially abundant proteins in the cerebrospinal fluid (CSF) between CIS patients who converted to MS and those who remained as CIS. Proteomic findings were validated by ELISA and CHI3L1 levels were significantly increased in the CSF of MS converters and high levels were associated with a shorter time to MS compared to non-MS converters^1^. Based on these initial promising results, our group conducted a validation study of CHI3L1 as prognostic biomarker in the CSF of 813 CIS patients from 15 MS centres also classified according to their conversion to MS based on clinical and radiological criteria^2^. In a multivariable Cox regression analysis including radiological abnormalities at baseline, presence of IgG oligoclonal bands, treatment, and age at CIS onset as covariates, patients with high CSF levels of CHI3L1 had a 4-fold increased risk for the development of neurological disability compared to CIS patients with low CSF CHI3L1 levels at the time of the first neurological event^2^. Moreover, high CSF CHI3L1 levels were associated with earlier disability progression (5-year difference as median time) compared to patients with low protein values and with sensitivity over 70%^2^. Interestingly, CSF CHI3L1 levels were associated with the development of brain atrophy evaluated by the brain parenchymal fraction change after 1 and 5 years from the CIS event^2^. Based on the above-mentioned findings of a strong association between high CSF CHI3L1 levels and the development of neurological disability and brain atrophy in CIS patients, the question that then arises is whether CHI3L1 is just a mere biomarker that can be measured in the CSF and reflects the degree of astrocyte activation, or CHI3L1 could be neurotoxic *per se*. Considering that neuronal damage is responsible for the permanent neurologic disability observed in MS patients^3,4^, in the present study we aimed to explore the potential neurotoxic effect of CHI3L1 by adding the protein at different concentrations *in vitro* to primary neuronal cultures.

## Results

### CHI3L1 impairs neuronal functionality and survival

**Figure 1:**
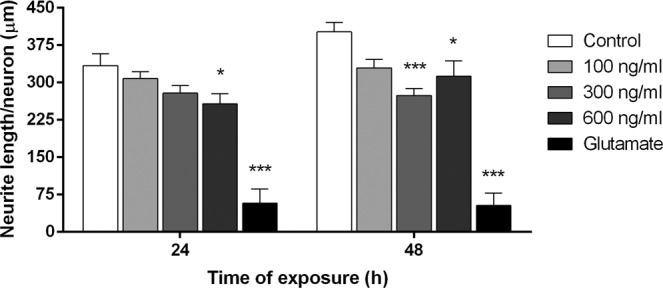
Dose-response study of CHI3L1 neurotoxic effect. Cortical neurons from E16 mice at 11DIV were treated with mouse recombinant CHI3L1 (100, 300 and 600 ng/ml), glutamate (200 µM) or medium (control) for 24 and 48 hours. Quantitative analysis of neurite length per neuron was performed with Image J plugin NeuriteTracer. CHI3L1 at 300 ng/ml induced a significant shortening of neurite length after 48 hours (p = 2 × 10^−5^) while 600 ng/ml showed a neurite length retraction both at 24 and 48 hours (p = 0.04 vs. control). Five fields (20x) of each replicate were randomly chosen, imaged and counted. Data are shown as mean ± standard error of the mean from 2 independent experiments with 4 replicates within each group. *p < 0.05, **p < 0.01, ***p < 0.001 (Statistics: one-way ANOVA).

A preliminary dose-response study was first conducted to determine the optimal exposure conditions of cortical neurons to CHI3L1. Different CHI3L1 concentrations below and above 170 ng/ml (100, 300 and 600 ng/ml), a cut-off value that was demonstrated to have prognostic implications in CIS patients^2^, were tested for 24 and 48 hours. A CHI3L1 concentration of 300 ng/ml was selected as the lowest dose that exhibited neuronal function impairment (Fig. 1; p = 2 × 10^−5^ vs. control) to test its potential toxic effect in primary neuronal cultures.

**Figure 2:**
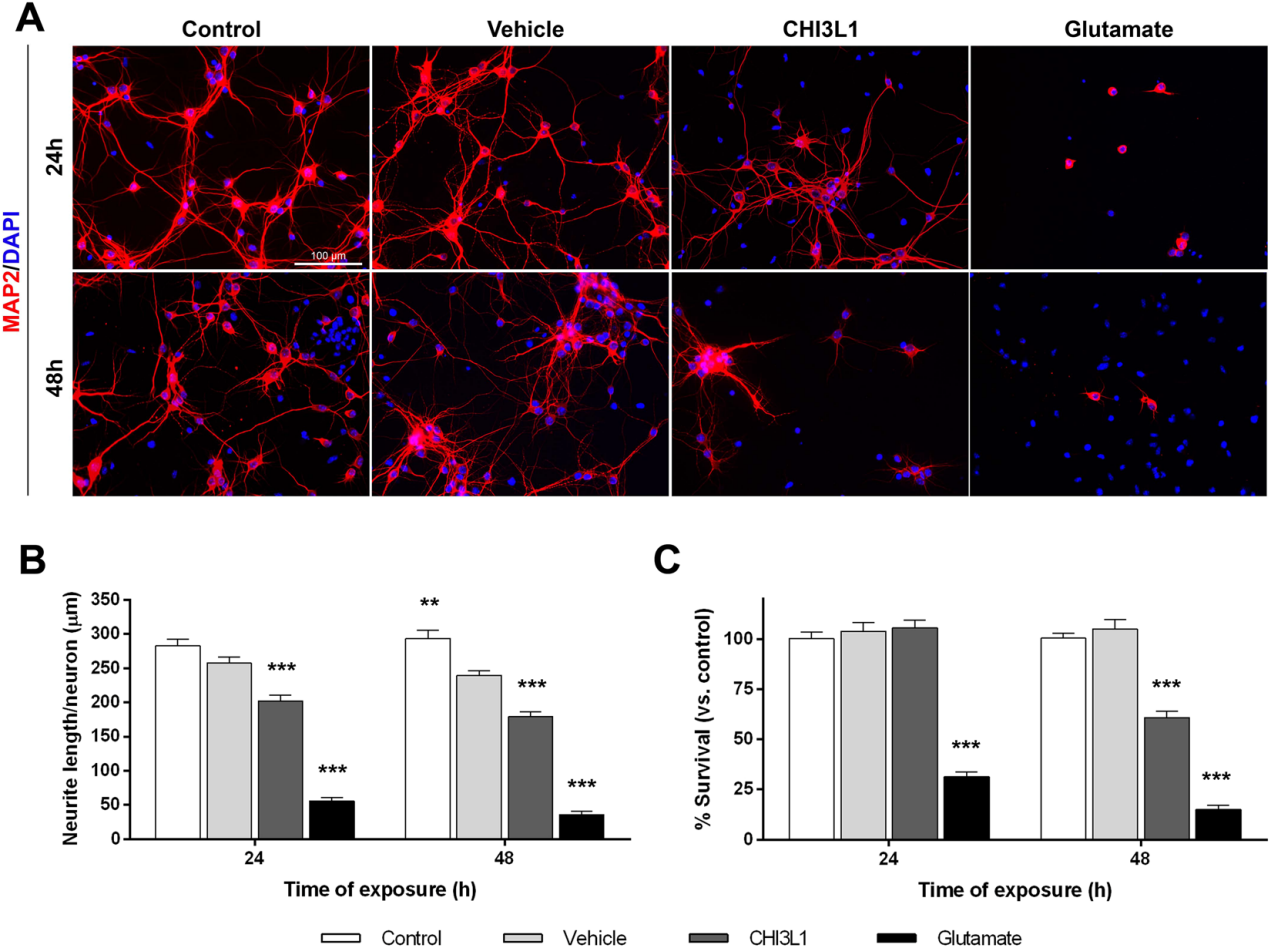
Neurotoxic effect of CHI3L1. Cortical neurons from E16 mice at 11DIV were treated with PBS (vehicle), mouse recombinant CHI3L1 (300 ng/ml), glutamate (200 µM) or medium (control) for 24 and 48 hours. (**A**) Representative images of neurons immunostained for MAP2 (red) and DAPI (blue). Scale bar, 100 µm. (**B**) Quantitative analysis of neurite length per neuron was performed with Image J plugin NeuriteTracer. CHI3L1 induced a significant retraction of neurite length per neuron after 24 (p = 0.0007) and 48 hours (p = 0.0001) compared to the vehicle. (**C**) Quantification of neuronal survival is expressed as the percentage of the mean total number of neurons respect to the control condition. After 48 hours, CHI3L1 significantly reduced neuronal survival (p < 0.0001 vs. vehicle). (**B,C**) Five fields (20x) of each replicate were randomly chosen, imaged and counted. Data are shown as mean ± standard error of the mean from 5 independent experiments (vehicle: n = 3) with 4 replicates within each group. *p < 0.05, **p < 0.01, ***p < 0.001 (Statistics: one-way ANOVA).

After 24 hours of treatment, assessment of neuronal functionality with the neuron-specific cytoskeletal protein MAP2 revealed that CHI3L1 significantly shortened the total neurite length per cell and induced a neurite length retraction of 21.5% (Fig. 2A,B; p = 0.0007 vs. vehicle). This neurotoxic effect was more prominent after 48 hours of treatment, exposure time at which CHI3L1 induced a 25.4% of neurite length retraction (Fig. 2A,B; p = 0.0001 vs. vehicle).

Evaluation of cell death showed that after 48 hours of treatment, CHI3L1 significantly decreased the total number of neurons and reduced neuronal survival by 44.0% (Fig. 2A,C; p < 0.0001 vs. vehicle), whereas no toxic effect was observed after 24 hours of exposure.

Glutamate, which was used as a positive control of excitotoxicity, significantly induced both neuronal function impairment and cell death following 24 and 48 hours of exposure compared to all experimental conditions (Fig. 2A–C).

### CHI3L1 does not impair survival of immune cells and other central nervous system (CNS) cells

**Figure 3:**
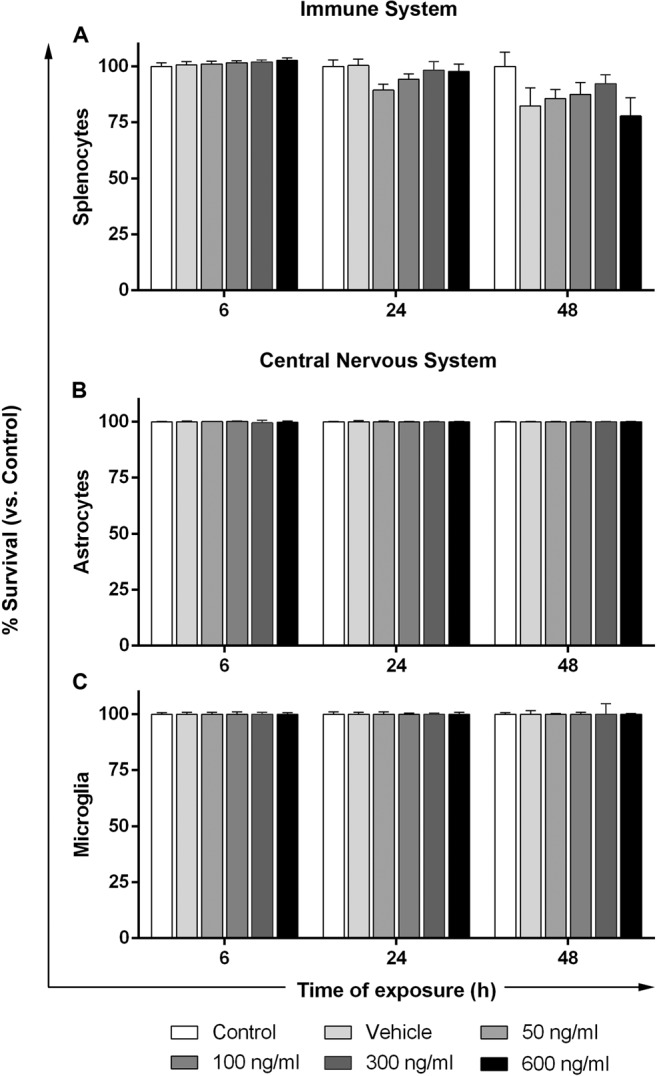
CHI3L1 effect on immune and CNS cells. Splenocytes,astrocytes and microglia from mixed glial cultures were treated with PBS (vehicle), mouse recombinant CHI3L1 (50, 100, 300 and 600 ng/ml) or medium (control) for 6, 24 and 48 hours. (**A**) Quantification of splenocyte survival is expressed as the percentage of CD45^+^ alive cells respect to the control condition. (**B**) Quantification of astrocyte survival is expressed as the percentage of parental alive cells respect to the control condition. (**C**) Quantification of microglia is expressed as the percentage of CD11b^+^ alive cells respect to the control condition. No significant effect on cell survival was observed in any of the doses, time points or cell types of the study. Data are shown as mean ± standard error of the mean from 5 independent experiments for splenocytes and from 3 independent experiments with duplicates within each group for astrocytes and microglia. (Statistics: one-way ANOVA).

In order to determine whether the cytotoxic effect of CHI3L1 was neuron-specific or could also be observed in other cell types, we treated splenocytes, as representative cells from the peripheral immune system, and astrocytes and microglia, as representative cells from the CNS, with different concentrations (50, 100, 300 and 600 ng/ml) of CHI3L1 *in vitro* for 6, 24 and 48 hours. As shown in Fig. 3, none of the experimental conditions were associated with a significant effect of CHI3L1 on cell death of immune or CNS cells.

## Discussion

CHI3L1 is a secreted glycoprotein member of the glycoside hydrolase 18 chitinase family that targets chitin but lacks enzymatic activity^5^. Within the CNS, CHI3L1 is mainly secreted by reactive astrocytes^2,6^, particularly in lesions with high inflammatory activity, but also by macrophages/microglial cells in lesions with low inflammatory activity^2^. A recent validation study conducted by our group in more than 800 CSF samples confirmed a role of CHI3L1 as prognostic biomarker in CIS patients^2^. The strong association observed in CIS patients between high CSF CHI3L1 levels and development of disability as an independent and unique risk factor^2^ prompted us to investigate a potential neurotoxic effect of CHI3L1. Exposure of primary cultures of mouse cortical neurons to CHI3L1 at a concentration of 300 ng/ml resulted in a significant reduction of neurite length and neuronal survival after 48 hours. This concentration is above the 170 ng/ml cut-off value that best discriminated in our study between high and low CHI3L1 levels in terms of development of disability^2^. In line with our results, a previous study focused on up-regulated genes in astrocytes expressing mutated TAR DNA binding protein 43 (TDP-43), a causative factor in amyotrophic lateral sclerosis, also showed decreased cell viability in cortical neurons treated at 10 DIV with CHI3L1 for 4 days, although at higher doses (500–1000 ng/ml)^7^. Interestingly, in our study, exposure of neurons to CHI3L1 was also associated with significant neurite retraction after 24 hours, indicating an early effect of CHI3L1 in neuronal functionality. In this context, it is widely known that dendritic structure and morphology closely correlate to neuronal function and health^8,9^.

The fact that CHI3L1 showed no effect on the survival of disease-involved immune cells, like splenocytes, and other CNS cells, such as astrocytes and microglia, which are the main CHI3L1-secreting cells, supports a specific neurotoxic effect rather than a generalized non-specific cytotoxic effect of CHI3L1. We focused on neurons since neuronal damage is the main responsible for the permanent neurologic disability observed in patients with MS^3,4^. However, an effect of CHI3L1 in oligodendrocytes cannot be ruled out, being a limitation of the study.

Overall, these results point to a neurotoxic effect of CHI3L1 *in vitro* and suggest a potential role of CHI3L1 as therapeutic target to prevent disability progression in MS patients.

## Methods

### Animals

Female (9–12 week-old) and pregnant (6–10 week-old) C57BL6/J mice, E16 C57BL6/J mouse embryos and P0–-1 postnatal C57BL6/J mouse pups were used for primary cultures. Procedures were conducted in accordance with the guidelines established by the Ethical Committee for the Use of Laboratory Animals in Spain (Real Decreto 53/2013; Generalitat de CatalunyaDecret 214/97) and the European Ethical Committee (Directive 2010/63/UE). All experimental protocols were approved by the Clinical Research Ethics Committee of the Institut de Recerca Valld’Hebron (VHIR).

### Primary neuronal cultures

Primary neuronal cultures were prepared from E16 cortices of C57BL6/J mouse embryos. Briefly, embryos were decapitated and brains were dissected out. Meninges, choroid plexus and hippocampus were removed and cortices were mechanically and enzymatically disrupted in the presence of trypsin and DNase I (Sigma) for 10 min at 37 °C. Cell suspensions were filtered through a 70-µm cell strainer and plated on coverslips precoated with 0.1 mg/ml poly-D-lysine (Sigma) in 24-well plates. Neurons were seeded at a density of 75,000 cells/cm^2^ in Advanced DMEM (Gibco) supplemented with 10% Fetal Bovine Serum (FBS, heat inactivated, Sigma), 20 U/ml penicillin and 20 µg/ml streptomycin (Gibco). Three hours after seeding, medium was replaced with serum-free Neurobasal medium (Gibco) containing 2% B27 supplement (Gibco), 1% GlutaMAX (100×, Gibco), 20 U/ml penicillin and 20 µg/ml streptomycin (Gibco). Culture medium was partially changed once per week with fresh supplemented Neurobasal medium. Cultures were maintained at 37 °C, in a humidified atmosphere containing 5% CO_2_ and treated with vehicle (PBS) or diverse concentrations of mouse recombinant CHI3L1 protein (R&D Systems) for 24 and 48 hours at 11 days *in vitro* (DIV). Glutamate (Sigma) at 200 µM was used as a positive control of neuronal excitotoxicity.

### Primary splenocyte cultures

Spleens from five euthanized female C57BL6/J mice were removed and cell suspensions were obtained by grinding the spleens through a 70-μm cell strainer. Splenocytes were seeded at 2 × 10^5^ cells/well into 96-well plates in X-VIVO15 medium (Lonza) supplemented with 1% v/v L-glutamine (Biowest), 0.4% v/v penicillin-streptomycin (Gibco), 0.1M HEPES (Gibco) and 6 μM 2-β-mercaptoethanol (Sigma).

### Primary astrocyte cultures

Purified primary astrocyte cultures were prepared from P0–1 cortices of C57BL6/J mouse pups. Cell suspensions were purified by magnetic cell separation (MACS) using CD11b MicroBeads (#130-093-634, Miltenyi Biotech) to specifically eliminate microglia from cell culture. Astrocytes were plated on 24-well plates precoated with 0.1 mg/ml poly-D-lysine (Sigma) and seeded at 35,000 cells/cm^2^ in Advanced DMEM (Gibco) supplemented with 10% FBS (Sigma), 20 U/ml penicillin and 20 µg/ml streptomycin (Gibco). The medium was changed every 3 days and cells were used for experiments at 7 DIV.

### Primary mixed glial cultures

Primary mixed glial cultures were prepared from P0–1 cortices of C57BL6/J mouse pups. Cells were plated on  24-well plates precoated with 0.1 mg/ml poly-D-lysine (Sigma) and seeded at 62,500 cells/cm^2^ in DMEM-F12 (Gibco) supplemented with 10% FBS (Sigma), 20 U/ml penicillin and 20 µg/ml streptomycin (Gibco). The medium was changed every 3 days and cells were used for experiments at 15 DIV.

### Immunofluorescence

Neurons were fixed in cold 4% paraformaldehyde for 20 min at room temperature. After washing with PBS, cells were permeabilized in 0.1% Triton X-100 in PBS for 15 min and blocked with 5% Normal Goat Serum (NGS, Millipore) in PBS for 1 h. Then, cells were incubated with mouse monoclonal anti-MAP2 diluted at 1:500 (microtubule-associated protein-2, #M1406, Sigma) in 1% NGS in PBS at 4 °C overnight. After three washes with PBS, the secondary antibody goat anti-mouse Alexa Fluor 594 diluted at 1:500 (#A11032, Invitrogen) in blocking solution was added. DAPI (4′,6-diamidino-2-phenylindole, 1:20000, Sigma) was used for nuclei staining and coverslips were mounted with Vectashield (Vector Laboratories). All images were acquired using a Leica DFC550 fluorescence microscope and LAS V4.5 visualization software (https://www.leica-microsystems.com/) with an objective magnification of 20×.

### Cell death and neurite tracing

The effect of CHI3L1 on both neuronal death and functionality was analysed on fluorescent microscopy images immunostained for DAPI and MAP2. Cell survival was assessed by quantifying the mean total number of neurons after treatment respect to the control condition.Neuronal functionality was determined using Image J (https://imagej.nih.gov/ij/) plugin NeuriteTracer (https://fournierlab.mcgill.ca/styled-6/NeuriteTracer.html)^10^. This method allows simultaneous automated nuclei counting and neurite tracing measurement by processing pairs of neuronal and nuclear marker images given user-defined thresholds and correction for non-specific background. Both quantifications were performed in 5 randomized fields per condition, each one performed in 4 replicates, from a total of 5 independent experiments (vehicle: n = 3). An average of 1,046 neurons from each condition (Control = 1,313, vehicle = 1,397, CHI3L1 = 1,081 and glutamate = 394) per independent experiment were analysed.

### FACS analysis

Splenocyte, astrocyte and mixed glial cultures were untreated or treated with vehicle (PBS) or diverse concentrations of mouse recombinant CHI3L1 protein (R&D Systems) for 6, 24 and 48 hours. The effect of CHI3L1 on cell death was assessed using Fixable Viability Stain 510 (BD Biosciences) and flourochrome-labeled anti-CD45 mAb (#561869, BD Biosciences) for splenocytes or Fixable Viability Stain 510 (BD Biosciences) and anti-CD11b mAb (#25-0112-81, eBioscience) for microglia. No lineage-specific marker was used for astrocyte analysis due to the purity of astrocyte culture. Samples were acquired with in a CytoFLEX (Beckman Coulter) flow cytometer and data analysis was performed with CytExpert 2.3 software (https://www.beckman.es/flow-cytometry/instruments/cytoflex/software).

### Statistical analysis

All values are expressed as the mean ± standard error of the mean (SEM). Data were analysed using R version 3.5.3 (https://www.r-project.org/) statistical software. Comparisons between groups were evaluated by one-way ANOVA with Tukey multiple comparison of means test. Outlier values were identified by the Grubb’s test and excluded from the analyses when applicable (α = 0.05).

## Data Availability

All data generated or analyzed during this study are included in this published article (and its supplementary information files).
